# Subfamily hypostominae: similarities and differences in testicular structure of amazonian fish

**DOI:** 10.1186/s40850-021-00106-5

**Published:** 2022-01-04

**Authors:** Ivana Kerly S. Viana, Gicelle M. F. S., Juliana C. D. Pantoja, Renata S. Oliveira, Yanne A. Mendes, José Leocyvan G. Nunes, Maria Auxiliadora P. Ferreira, Rossineide M. Rocha

**Affiliations:** 1 Laboratory of Cellular Ultrastructure, Institute of Biological Sciences, Federal University of Pará, Belém, Pará, 66075-110 Brazil; 2 Laboratory of Immunohistochemistry and Developmental Biology, Institute of Biological Sciences, Federal University of Pará, Belém, Pará, 66075-110 Brazil

**Keywords:** Loricariidae, Amazonian fish, Testis, Spermatogenesis, Histology

## Abstract

**Background:**

Hypostominae is a subfamily of the family Loricariidae that has a great diversity of species. Accordingly, testicular studies in fish can contribute to the phylogeny and taxonomy of species and to the comparison of reproductive aspects between species. Thus, this work aimed to characterize the testicular morphology and spermatogenesis of the *Hypostominae* species *Baryancistrus xanthellus*, *Peckoltia oligospila* and *Hypancistrus zebra*.

**Results:**

*B. xanthellus*, *P. oligospila* and *H. zebra* had an anastomosed tubular type of testis. The germinal epithelium was continuous with unrestricted spermatogonia, and the proliferative, meiotic and spermiogenic phases were defined in all species. In the spermiogenic phase, spermatids were classified as initial, intermediate and final. Only in *B. xanthellus* in the final phase was there nuclear rotation. The sperm for the three species had a head with an oval shape and a single flagellum composed of the short midpiece, principal piece and end piece. *B. xanthellus* and *P. oligospila* showed a cylindrical flagellum and *H. zebra* showed projections that produced a flattened appearance.

**Conclusions:**

On the basis testicular structure and ultrastructural characteristics of the germ cells, there was a greater relationship between *B. xanthelus* and *P. oligospila*, while *H. zebra* had particular characteristics. These aspects show that despite belonging to the same subfamily, the species have distinct biological characteristics.

## Background

For several decades, knowledge of the testicular structure and spermatogenesis of fish has been important to understand the reproductive biology of the species and the mechanisms involved in the maturation of sperm[1–3]. More recently, these data have established different reproductive strategies, evolutionary adaptations to aquatic environments[4–7] and a broad understanding of phylogenetic and systematic relationships [8, 9]. However, there are few studies that address the morphological differences between species of the same family [9–11], or between subfamilies [12, 13]. These characteristics can help to understand the relationships between species and support the permanence of a species in a family or its removal.

In general, fish testes can be classified according to morphology as filiform, fringed or lobular [4] and as types regarding the organization of the germinal epithelium, i.e., anastomosed tubular when seminiferous tubule anastomosis occurs, and lobular because the epithelium is in a closed compartment and because the seminiferous tubules end at the periphery of the testis [14]. However, during the development of the germinal epithelium, spermatogenesis is subdivided into three phases: proliferative, where there are successive mitotic divisions of type A and B spermatogonia; meiotic, where primary and secondary spermatocytes pass through meiotic divisions giving rise to spermatids; and spermiogenesis where spermatids give rise to spermatozoa [15].

The family Loricariidae is endemic to South America and widespread in the Amazon region, and it is made up of many species that are exploited due to their high value in the aquarium market [16]. This family is subdivided into the subfamilies Delturinae, Hypoptopomatinae, Hypostominae, Lithogeninae, Loricariinae and Neoplecostominae, where Hypostominae stands out because it contains a greater number of species, ecologically and morphologically diverse, which hinders a more comprehensive phylogeny revision [17]. The main studies that address the morphological differences in this subfamily have focused on the external anatomy of the digestive tract and osteology [18–20], and there is no information as to whether the testicular characteristics represent a relationship between species. Among the species of this subfamily are: *Baryancistrus xanthellus* Rapp Py-Daniel et al. 2011, *Peckoltia oligospila* Günther 1864 and *Hypancistrus zebra* Isbrücker & Nijssen 1991. All have a high value in the ornamental fish market, but *H. zebra* is the species that stands out for being on the Red List of species threatened with extinction, which confers greater vulnerability. This is even more so because of the anthropic influence in the Amazon region, such as the establishment of hydroelectric dams, which cause damage to natural habitats and interfere with the life cycle, resulting in a great loss of species diversity [21].

Although they are species of the same subfamily, there is no information as to whether there are differences or similarities in testicular structure and organization of the germ cells. These basic biology parameters are of fundamental importance because they help to understand the life history of the species [22]. On the basis of this thinking, the present study aimed to compare the testicular structure and spermatogenesis of three species belonging to the subfamily Hypostominae, namely *B. xanthellus*, *P. oligospila* and *H. zebra*, to establish the proximity relations.

## Results

### Testis morphology

**Fig. 1:**
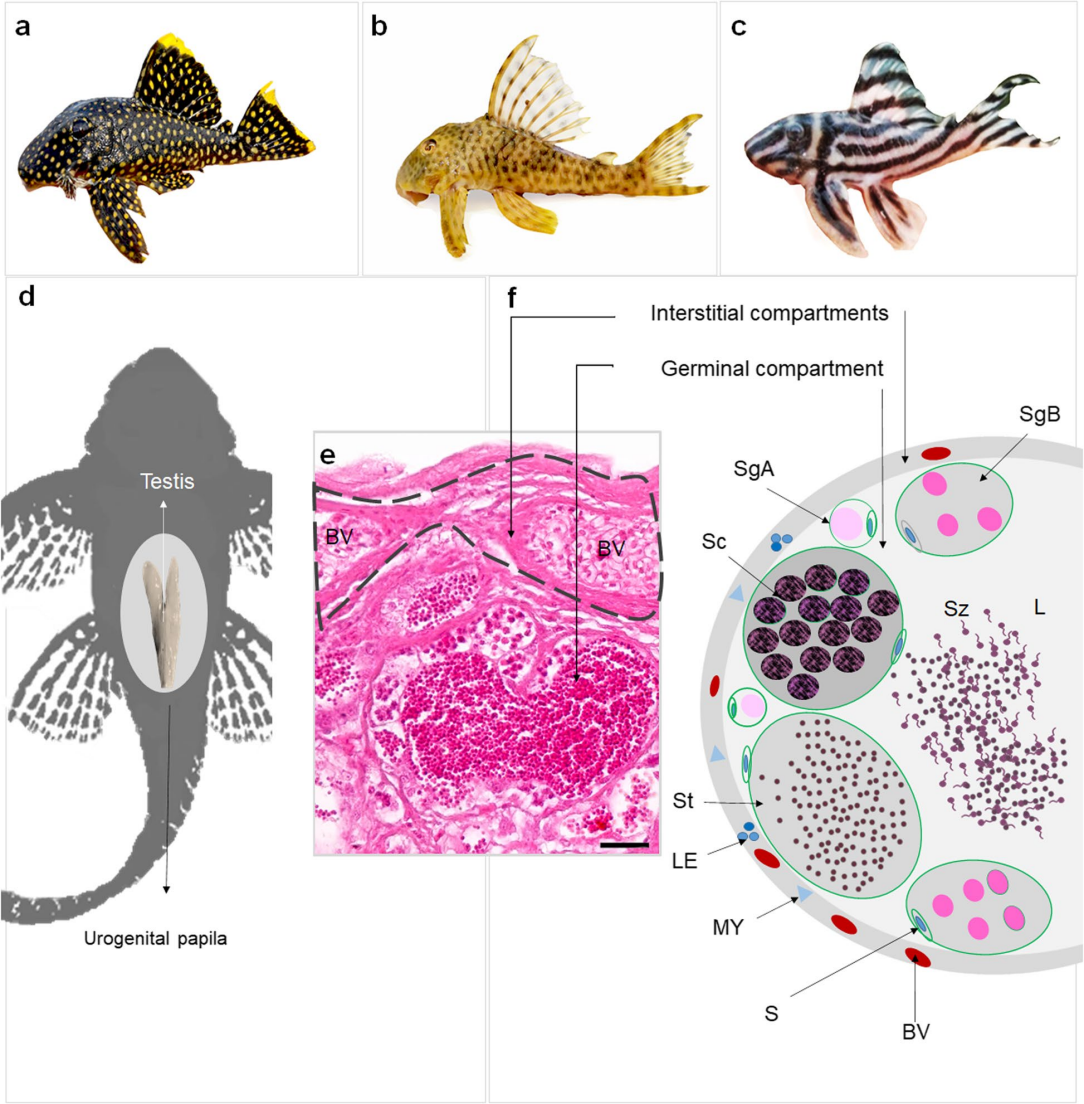
species morphology and testicular structure. **a**,**b**,**c** External morphology of the species; **d** Schematic representation of the testicle of the species where the organ is paired and forms a spermatic duct that leads to the urogenital papilla; **e** Photomicrography of the mature *B. xanthellus* gonad showing germinal and intestinal compartments Scale bar: 25 μm; **f** Schematic representation of the interstitial compartment containing blood vessels (BV), myoid cells (MY), interstitial or Leydig (LE) cells. Seminiferous tubules formed by germinative compartment, containing Sertoli cells (S), spermatogonia (SgA/SgB), spermatocytes (Sc), spermatids (St) and spermatozoa (Sz)

Macroscopically, the testes of the species *B. xanthellus*, *P. oligospila* and *H. zebra* (Fig. 1a-c), showed a color that varied from transparent to whitish. The testes were paired, lobular organs of equal size, and they were fused caudally, forming a single sperm duct leading to the urogenital papilla (Fig. 1d). The organ was covered by the tunica albuginea, which produces septa in the organ, forming two compartments. The interstitial compartment had blood vessels, myoid cells, interstitial or Leydig cells and connective tissue. The germinal compartment consisted of two cell types, Sertoli or somatic cells and germinal cells (spermatogonia, spermatocytes, spermatids and spermatozoa) (Fig. 1 e,f).

**Fig. 2:**
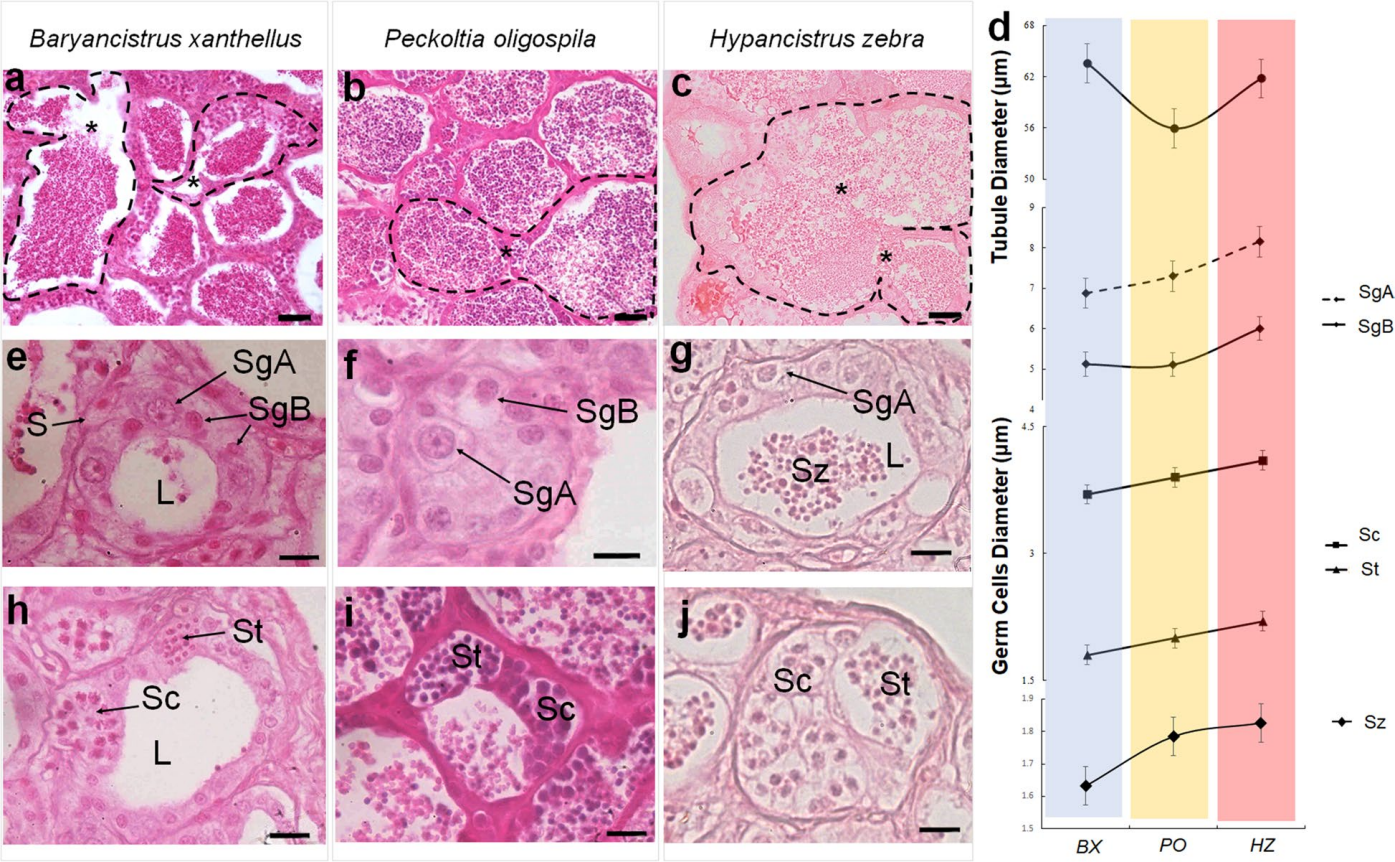
Analysis and morphometry of the germ cell and seminiferous tubule. **a**,**b**,**c** Testis with anastomosed (*) seminiferous tubules (dotted line); **d** Mean and comparative diameter of the seminiferous tubules of A spermatogonia (SgA), B spermatogonia (SgB), spermatocytes (Sc) and spermatids (St) between the species *B. xanthellus* *(BX)*, *P. oligospila* *(PO)* and *H. zebra* *(HZ)*; **e**,**f**,**g**,**h**,**i**,**j** Photomicrography of the seminiferous tubule, showing spermatogonia (SgA, SgB), spermatocytes (Sc) and spermatids (St), and spermatozoa (Sz) in the tubule lumen (L). Scale bar: a-c:25 μm, e-j: 10 μm

In the mature stage, *B. xanthellus, P. oligospila* and *H. zebra* showed testicular organization of the tubular anastomosis type, where the tubules are previously separated and then anastomosed (Fig. 2a-c).

According to the size of the seminiferous tubules, *B. xanthellus* had the largest diameter (174.37 ± 70 μm), followed by *H. zebra* (102.97 ± 33.68 μm) and *P. oligospila* (101.54 ± 46.59 μm), and significant differences were observed only between *H. zebra* and *P. oligospila* (H = 209.23; df = 2; *p* > 0.05) (Fig. 2d). The proliferative, meiotic and spermiogenic phases were defined according to the organization of the germinal epithelium.

### Proliferative phase

**Fig. 3:**
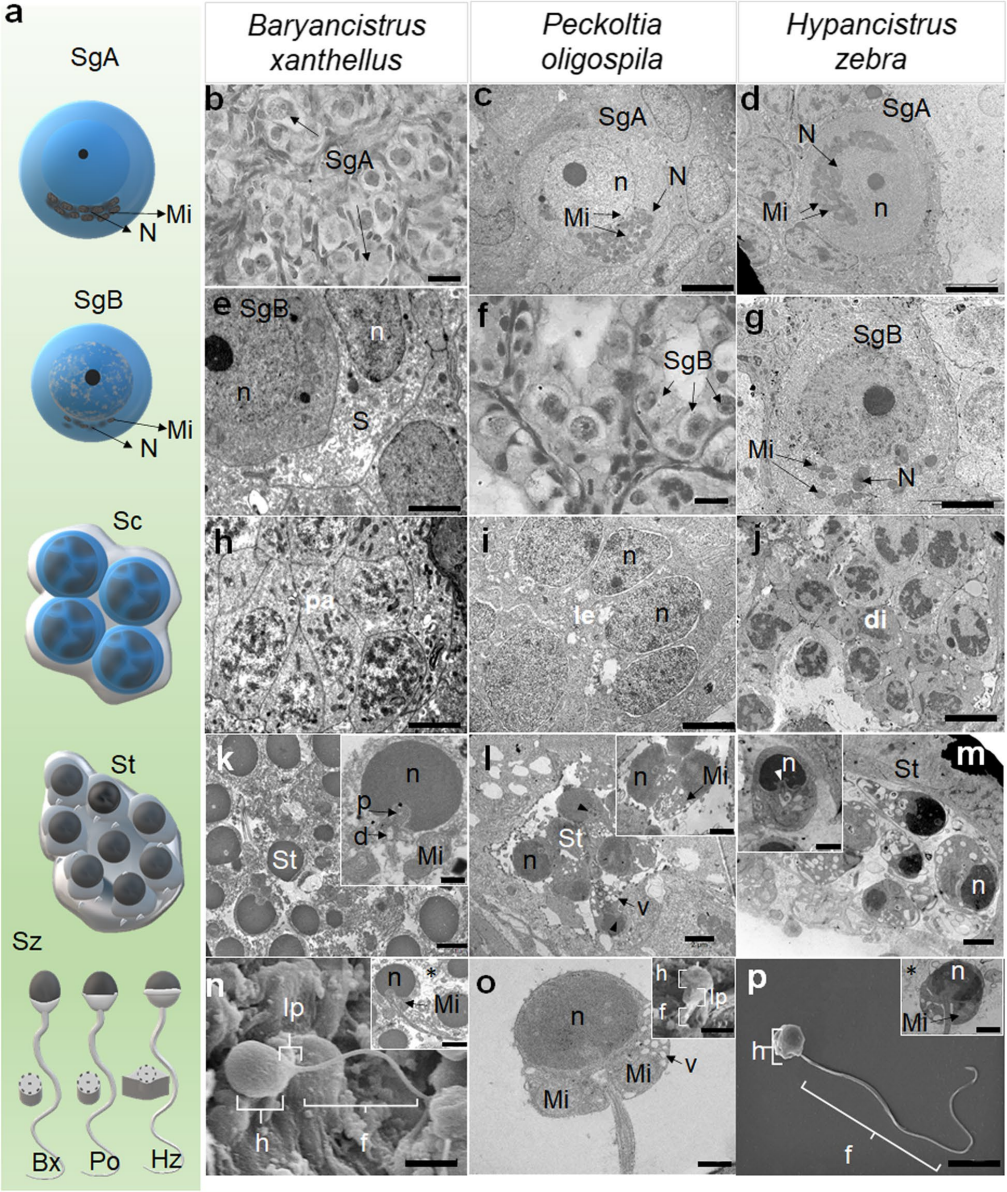
Photomicrography of spermatogenic cells during the germinative phases. **a,b,c,d,e,f,g** Proliferative phase: **a** schematic design demonstrating the similarities of spermatogenesis between the species and differences in the spermatozoa; **b**,**c,d** spermatogonia (SgA) with mitochondria (Mi) and nuages (N); **e**,**f**,**g** B spermatogonia (SgB) forming cell nest and Sertoli cell (S) with presence of nucleus (n); **h,i,j** meiotic phase; **h** spermatocyte in pachytene (pa); **i** spermatocyte in leptotene (le); **j** spermatocyte in diplotene (di); **k,l,m,n,o,p** spermiogenic phase; **k** final spermatids (St), in detail, the presence of the proximal (p) and distal (d) centrioles and mitochondria (Mi); **l** initial spermatids (St), nest cell, nucleus (n), vesicles (v) and mitochondria (Mi); **m** intermediate spermatids with spherical nucleus (n); note intermediate spermatid showing deep nuclear fossa (arrowhead); **n**,**o**,**p** spermatozoa with head (h), midpiece (Ip) and flagellum (f),secretion (*); note, longitudinal view of Sz, showing a nuclear fossa; spermatozoa with oval nucleus (n), detail of head (h). Scale bar: light microscopy-b,f: 10 μm; TEM- c-e, g-j: 5 μm, k-m: 2 μm, insert: 500 nm; SEM—n,p: 2 μm, insert: 1 μm

When analyzing the germinal epithelium of *B. xanthellus*, *P. oligospila* and *H. zebra*, a continuous epithelium was observed with the presence of Sertoli cells and spermatogenic cells: spermatogonia, spermatocytes, spermatids and spermatozoa (Fig. 2e-j). Sertoli cells showed the characteristics of spermatogenic cells, forming spermatic cysts within the seminiferous tubule (Fig. 2e); they had an elongated body and irregular electron-dense nucleus (Fig. 3e).

Spermatogonia were observed as two types, A and B, in all species, and were not restricted to a single position in the seminiferous tubule, characterizing an unrestricted spermatogonial epithelium (Fig. 3 a-g). Type A spermatogonia (SgA) were observed isolated in the germinal epithelium (Fig. 3 a-d). Ultrastructurally, they had a clear cytoplasm, with the presence of mitochondria and electron-dense structures known as nuages close to the nuclear envelope, and they had a nucleus with a condensed chromatin and a single nucleolus (Fig. 3c, d). In relation to the nuclear envelope, this structure was more evident in *P. oligospila* (Fig. 3c). The cell diameter of SgA in *B. xanthelus*, *P. oligospila* and *H. zebra* was 6.59 ± 0.97, 7.59 ± 0.86 and 8.21 ± 0.92 μm, respectively, and significant differences were observed between the three species. (H = 511.71; df = 2; *p* < 0.05) (Fig. 2d).

Type B spermatogonia (SgB) appeared smaller and formed cell nests, and the cytoplasm had few mitochondria. The nucleus contained condensed chromatin with heterochromatin adhered to the nuclear envelope, and a central or peripheral nucleolus (Fig. 3 e–g), and these spermatogonia showed a cell diameter for *B. xanthelus*, *P. oligospila* and *H. zebra* of 4.99 ± 0.54, 5.75 ± 0.67 and 6.05 ± 0.56 μm, respectively, with significant differences being observed between the three species. (H = 297,13; df = 2; *p* < 0.05). Overall, for spermatogonia A and B, *H. zebra* displayed the largest spermatogonia (Fig. 2d).

### Meiotic phase

The primary spermatocytes formed nests near the wall of the seminiferous tubules, which are seen in different phases of prophase I (Fig. 3 h-j): leptotene is characterized by an ovoid nucleus with little condensed chromatin, showing some points of compaction and cytoplasm with little delimitation (Fig. 3i); in pachytene, the delimitation of the nucleus and nucleolus is not visible, and the condensed chromatin has a filamentous appearance (Fig. 3h); and in diplotene, chromosomes appear compacted with spots evident in the nucleus (Fig. 3j). Secondary spermatocytes were not seen. The diameter of the spermatocytes in *B. xanthelus*, *P. oligospila* and *H. zebra* was 3.92 ± 0.45, 4.35 ± 0.43 and 4.43 ± 0.47 μm, respectively, with no differences being observed between *H. zebra* and *P. oligospila* (H = 283.15; df = 2; *p* > 0.05) (Fig. 2d).

### Spermiogenic phase

In the three species, spermatids showed specific cellular characteristics that revealed a subdivision into phases, i.e., initial, intermediate and final (Fig. 3 k-m). The initial spermatids formed cysts, in which the cells are connected by cytoplasmic bridges, and have a spherical nucleus, with condensed chromatin and nuclear fossa, and cytoplasm with vesicles and mitochondria (Fig. 3l). The intermediate ones had a nucleus with condensed chromatin with electron-lucid regions and nuclear fossa, and there was little cytoplasm (Fig. 3m). In the final spermatids, the nucleus was spherical, and there was sparse cytoplasm and mitochondria in the midpiece. In this phase, nuclear rotation was observed only in *B. xanthellus*, and the proximal centriole was located in the nuclear fossa, while the distal centriole was perpendicular to the proximal one (Fig. 3k). The diameter of the spermatids in *B. xanthelus*, *P. oligospila* and *H. zebra* was 1.81 ± 0.29, 2.09 ± 0.20 and 2.21 ± 0.22 μm, respectively, where significant differences were observed between the three species. (H = 302,15; df = 2; *p* < 0.05) (Fig. 2d).

**Fig. 4:**
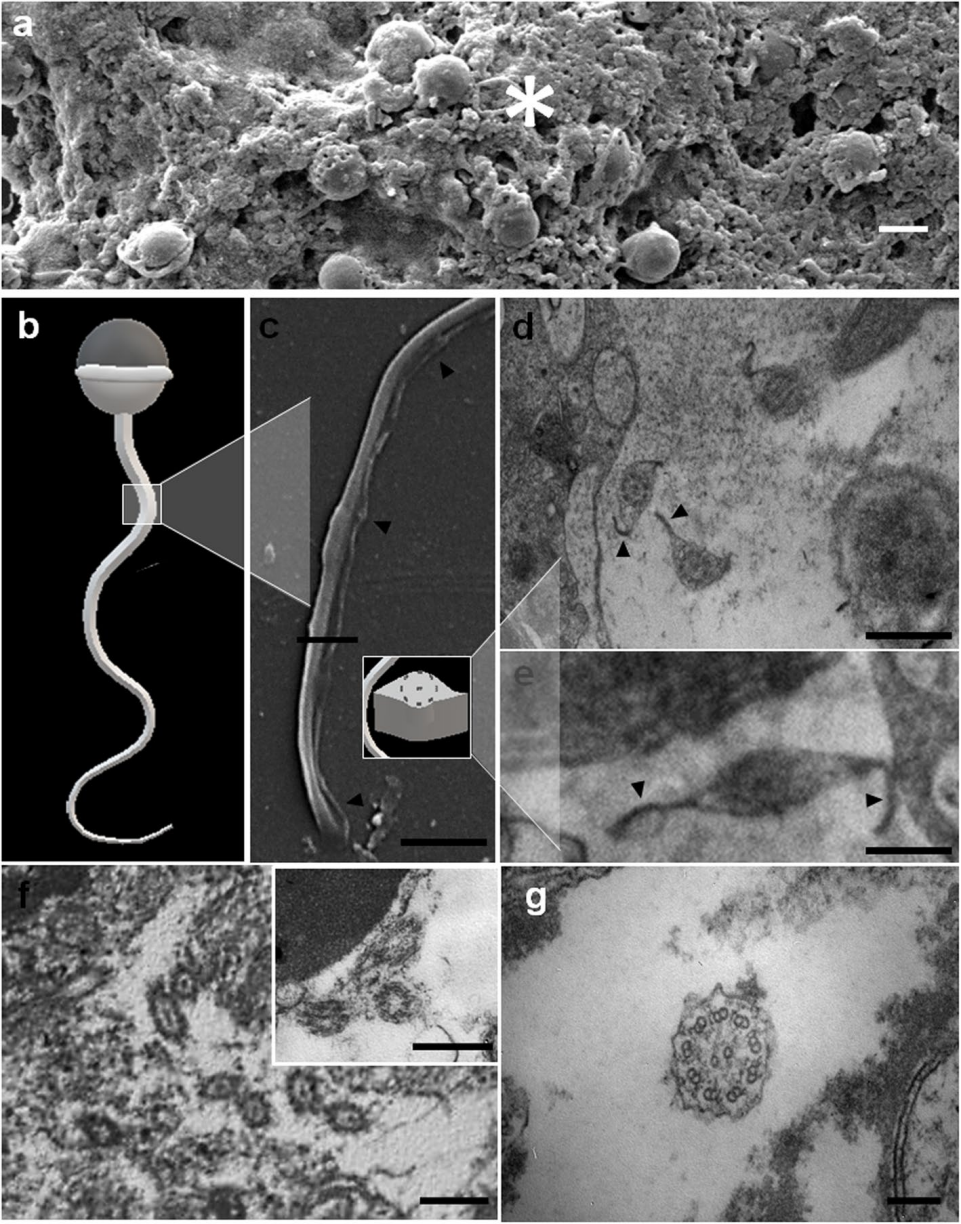
Photomicrography of sperm in the tubule lumen. a-e *H. zebra*: **a** Sperm immersed in secretion (*); **b** schematic design of sperm; **c** detail of the flagellum showing lateral projections (arrowhead) by SEM; **d** and **e** detail of the cross-section of the flagellum showing the lateral projections(arrowhead). **f** *B. xanthellus* **g** *P. oligospila*. Detail of the cross-section of the flagellum in both species. Scale bar: SEM- a: 2 μm, c: 3 μm; TEM- d,e: 1 μm

The spermatozoa were characterized as having compact chromatin, absence of acrosome, loss of residual bodies and formation of a single flagellum (Fig. 3 n-p). In *B. xanthellus*, the head of the spermatozoa was oval, with a single flagellum consisting of a short midpiece with the presence of mitochondria, long principal piece and short end piece (Fig. 3n). In *P. oligospila*, the nucleus had an oval shape and showed the presence of electron-lucid zones, and the midpiece was elongated and possessed mitochondria, while the principal piece was cylindrical (Fig. 3o). In *H. zebra*, the nucleus was half-sphere shaped and showed electron-lucid regions, and the midpiece appeared bulky and possessed mitochondria (Fig. 3p), while the principal piece displayed lateral projections which gave it a flattened shape (Fig. 4 b-e). In *P. oligospila* and *B. xanthellus* lateral projections were not observed in principal piece of the flagellum (Fig. 4f, g) In all species, the principal piece of the flagellum showed a 9 + 2 axonemal pattern and the end piece 9 + 0 pairs of microtubules (Fig. 3a). TEM and SEM demonstrated that the sperm of *B. xanthellus* (Fig. 3n: insert) and *H. zebra* (Fig. 3p: insert and 4a) were immersed in a secretion. The diameter of the spermatozoa head in *B. xanthelus*, *P. oligospila* and *H. zebra* was 1.63 ± 0.13, 1.78 ± 0.13 and 1.82 ± 0.12 μm, respectively, and significant differences were observed between the three species. (H = 168.28; df = 2; *p* < 0.05). Overall, *H. zebra* had the largest spermatozoa head (Fig. 2d).

**Fig. 5:**
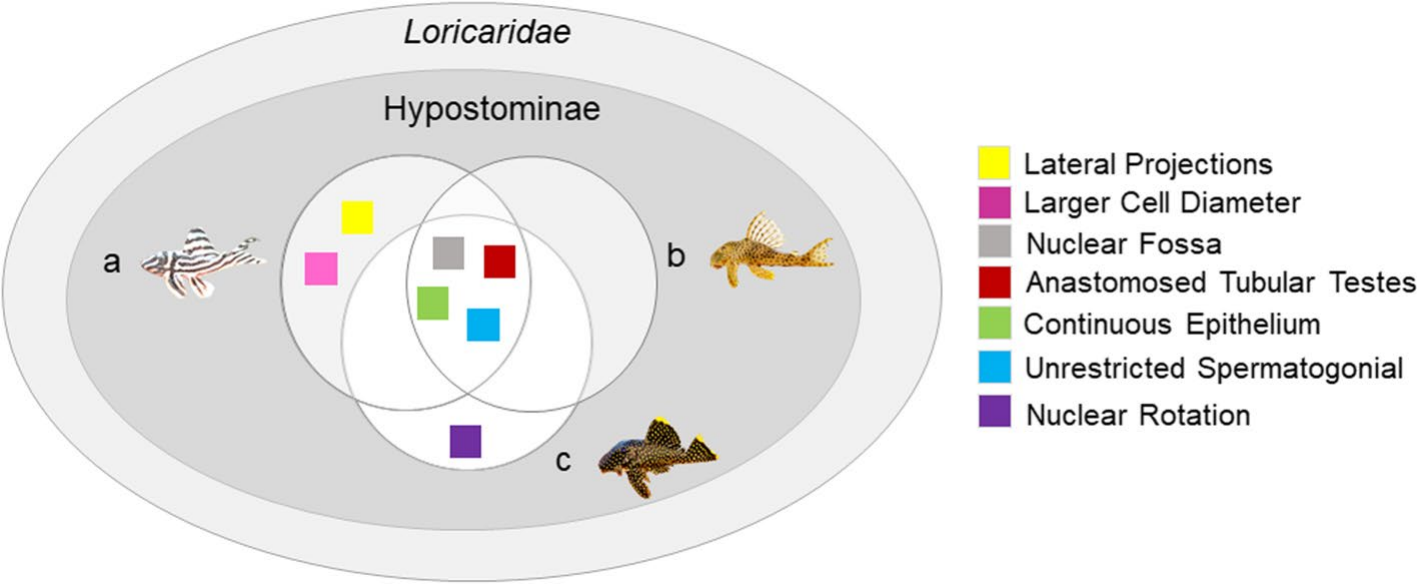
Relation of loricarid species based on differences and similarities in organization of germinative cells. **a ***H. zebra;* **b ***P. oligospila;* **c** *B. xanthellus* (C)

The testicular structure and the organization of the cells established the similarities and differences between the species studied in the subfamily (Fig. 5).

## Discussion

In this study, we compared testicular structure and spermatogenesis in three species of the family Loricariidae, subfamily Hypostominae. Despite the morphological diversity of testicular structures among the neotropical teleosts [23], *B. xanthellus*, *P. oligospila* and *H. zebra* have paired, lobular or saculiform testes, with color varying according to the gonadal stage. These characteristics are similar to those observed in the family Loricariidae [24–26].

The testicular structure for all species is of the anastomosed tubular type. This observed testicular structure is considered to be more basal within the phylogenetic classification of Teleostei [14]. Anastomosed tubular has also been seen in *Pterygoplichthys disjunctivus* [25]*, Hypostomus francis*ci [27], and *Harttia torrenticola* [28], species that belong to the order Siluriforme, family Loricariidae.

Here, we emphasize that the development of the germinal cells of the three species occurs within cysts present throughout the seminiferous tubule. This characteristic considered as continuous epithelium is in accordance with the literature [29] and has been observed in other species of the family Loricariidae, belonging to different subfamilies: Loricariinae (*Loricaria lentiginosa* [30] and *Harttia torrenticola* [28]), Hypoptopomatinae (*Pseudotocinclus tietensis* [31], and Hypostominae (*Hypostomus francisci* [27]. It is worth mentioning that this characteristic of the epithelium varies according to the degree of development of the testis, where a species can have both types of epithelium as seen in *Rachycentron canadum* (L.) [32], *Padogobius martensi* [33] and *Serrasalmus spilopleura* [8]. We believe that this characteristic is related to the capacity to renew the gametogenic lineage; considering the species studied, because they have a continuous epithelium, it is possible to observe in the whole reproductive cycle the presence of germinal cells occupying the entire wall of the seminiferous tubule.

In the present study, types A and B spermatogonia differed in size between species, where the largest diameter was found in *H. zebra.* Although the spermatogenesis process is similar among fish, characteristics such as cell diameter differ between species, and in the case of the subfamily Hypostominae, this difference is even more evident when compared with species such as *Pterygoplichthys disjunctivus*, which have A spermatogonia with a diameter of 14.87 ± 0.22 μm [25] and *Hypostomus francisci* with a diameter of 8.2 ± 1.1 µm [27]. Possibly this characteristic may be associated with the reproductive biology of each species.

We found that *B. xanthelus, P. oligospila* and *H. zebra* had a distribution of spermatogonia along the seminiferous tubule, being characterized as unrestricted spermatogonial. According to the literature [15], this type is common in anastomosed testes, whereas restricted spermatogonia are often seen in lobular testes. Only primary spermatocytes with chromatin at different levels of condensation have been observed. Similar results have been reported for other teleosts, showing that at this stage of division, secondary spermatocytes are difficult to visualize due to rapid cell division [5, 34].

In spermiogenesis, we showed peculiarities in spermatid development. The nuclear fossa was evident in *B. xanthellus, P. oligospila* and *H. zebra*. The presence of the nuclear fossa was observed in the Loricariidae in the subfamilies Hypoptopomatinae (*Hypoptopoma guentheri and Schizolecis guntheri*), Neoplecostominae (*Kronichthys heylandi and Neoplecostomus paranensis*) and Loricariinae (*Farowella oxyrryncha and Loricaria lata*) [13]. This suggests that most of the members of the family Loricariidae have this biological characteristic.

In general, the spermatids from *B. xanthelus*, *P. oligospila* and *H. zebra* also showed similarities and differences related to the head and flagellum. In the observations reported in literature [35], characteristics such as round or oval head, short midpiece with few mitochondria and single or double flagellum are observed in the spermatozoa of the aquasperm type, considered primitive, adapted to external fertilization. In most fish with external fertilization, spermatozoa without an acrosome penetrates the oocyte, which is facilitated by the presence of the micropyle [36–38].

The flagellum had a standard 9 + 2 microtubular arrangement, but in *H. zebra*, lateral projections were observed, which gave the flagellum a flatter shape. These projections have also been observed in other species of loricariids [13]. The morphology of the flagellum as well as its molecular structure accounts for spermatic motility [39], and differences in spermatid morphology are useful tools for systematic analysis of different habitats and reproductive modes in fish [34]. We believe that these lateral projections help in the efficiency of motility in relation to energy expenditure, in view of the different characteristic habitats of the species. As an example, spermatozoa of *H. zebra* have intermediate vigor and a long sustained motility that provides less energy expenditure [40]. This condition can help in reproduction since this species is endemic to the Xingu River and lives in stream rapids.

A relevant fact was the presence of a secretion in the lumen of the seminiferous tubules of *H. zebra* and *B. xanthelus*, which made it difficult to visualize the plasma membrane of these sperm. This result differs from *Pterygoplichthys disjunctivus* [25], subfamily Hypostominae. However, for other Loricariidae subfamilies such as Hypoptopomatinae and Otothyrinae [13], the same difficulty in visualization the sperm membrane has been reported due to the presence of this secretion. According to the literature [41], these substances for fish can assist in the fertilization process and in increasing seminal volume.

## Conclusions

On the basis of gonadal morphology, testicular structure, spermatozoa location and germ cell ultrastructural characteristics, we found that there is a greater relationship between *B. xanthelus* and *P. oligospila*. *H. zebra* has characteristics that differ from the others, although they are in the superorder Ostariophysia, in the same subfamily (Hypostominae) and are endemic species in the Amazon region. These results contribute to the understanding of spermatogenesis of neotropical species, Therefore, these findings are important for understanding the phylogeny of this very diverse group for enabling biotechnological studies such as cryopreservation of semen and induced reproduction of species in captivity, to guarantee a better strategy to survive in their environment.

## Methods

### Sample collection

The samples were collected between January 2019 and July 2019 in the middle Xingu River (3°12′52″ S, 51°11′23″). We collected 30 specimens of each species, namely *B. xanthellus, P. oligospila*, *H. zebra,* with the help of local fishermen, who sampled the fish by scuba diving. Captured fish were anesthetized with benzocaine hydrochloride (0.1 g.L^–1^) and euthanized with sodium pentobarbital solution (60–100 mg/kg). Subsequently, gonads were removed by ventral incision.

### Light microscopy

Fragments of gonads in the maturing and mature stage of *B. xanthelus, P. oligospila* and *H. zebra* were fixed in Bouin’s solution for 24 h. The samples were then dehydrated in increasing concentrations of ethanol, cleared in xylene and infiltrated and embedded in paraffin [42]. Sections 5 μm thick were cut and stained with hematoxylin and eosin (HE), and stained sections were examined under a Carl Zeiss light microscope (AxioStar Plus 1,169,151).

### Morphometry and statistical analysis

For morphometry only, testes in the mature stage of *B. xanthelus, P. oligospila* and *H. zebra* were evaluated, because this stage has the largest seminiferous tubules. A total of five specimens/species were analyzed, and for each specimen, the mean diameter of the 58 seminiferous tubules were measured. In each specimen, the mean diameter (*n* = 200) for each germ cell type (spermatogonia A and B, spermatocytes, spermatids and spermatozoa) were analyzed. Only cells that contained a nucleus were measured. Serial sections were cut, and the slides were evaluated under a photomicroscope with the software NIS-elements BR (4.00.07-bit), and measurements were made at 40X magnification. Each cell was overlaid with two dashed lines crossing at right angles in the middle of the cell, and the length of the segment of the line over that diameter of the cell was measured. The mean length of the two measurements was taken as the approximate diameter of the cell. Means were assessed for normality using the Shapiro–Wilk test and analyzed using the Kruskal–Wallis test (*P* < 0.05) (Zar, 1999). All analyses were performed using the R Development Core Team Program (2016).

### Transmission electron microscopy (TEM) and scanning electron microscopy (SEM)

Fragments of testis were fixed in Karnovsky’s solution (4% paraformaldehyde, 2% glutaraldehyde in 0.1 M sodium cacodylate buffer, pH 7.4) for 24 h. After fixation, the fragments were washed in 0.1 M sodium cacodylate buffer, pH 7.4 and post-fixed in 1% osmium tetroxide in 0.1 M sodium cacodylate buffer, pH 7.4 for 2 h. For TEM analysis, the fragments were dehydrated in an ascending acetone series and embedded in Epon 812. Ultra-thin sections were cut in a microtome and contrasted with uranyl acetate and lead citrate, followed by examination in a JEOL (JEM -100CX II) electron microscope. For SEM analysis, testicular sections and 500-µl aliquots of raw semen were fixed in Karnovsky’s solution and Then, a drop of the fixed semen sample was placed on a poly-L-lysine-coated coverslip.The samples were dehydrated in a graded ethanol series (30 to 100%) and critical-point dried using CO2 and mounted on stubs, coated with gold and examined using a LEO 1430 SEM.

## Data Availability

The datasets used and/or analyzed during the current study are available from the corresponding author on reasonable request.

## Abbreviations

BV: Blood vessels
LE: Leydig cells
L: Lumen
Mi: Mitochondria
MY: Myoid cells
N: Nuages
n: Nucleus
S: Sertoli cells
Sc: Spermatocytes
pa: Spermatocyte in pachytene
le: Spermatocyte in leptotene
di: Spermatocyte in diplotene
SgA: Spermatogonia A
SgB: Spermatogonia B
St: Spermatids
Sz: Spermatozoa
h: Head
Ip: Midpiece
f: Principal piece
v: Vesicles
